# Efficacy of Filgotinib in Moderate to Severe Ulcerative Colitis: A Prospective Study Using Partial Mayo Score, Ulcerative Colitis Endoscopic Index of Severity, and Geboes Histopathology Score

**DOI:** 10.1093/crocol/otaf030

**Published:** 2025-04-15

**Authors:** Yoshiyuki Shirouzu, Hideki Ishibashi, Masayoshi Kage, Yutaro Mihara, Yuka Sakakibara, Kazuyoshi Nagata, Asami Suzuki, Toshihiro Ohmiya, Tomoko Irie, Yasumi Araki, Keiichi Mitsuyama, Hidetoshi Takedatsu, Toshihiro Noake

**Affiliations:** Kurume Coloproctology Center/Kurume Hospital, Kurume-City, Japan; Kurume Coloproctology Center/Kurume Hospital, Kurume-City, Japan; Division of Pathology, Department of Medicine, Kurume University School of Medicine, Kurume-City, Japan; Division of Pathology, Department of Medicine, Kurume University School of Medicine, Kurume-City, Japan; Kurume Coloproctology Center/Kurume Hospital, Kurume-City, Japan; Kurume Coloproctology Center/Kurume Hospital, Kurume-City, Japan; Kurume Coloproctology Center/Kurume Hospital, Kurume-City, Japan; Kurume Coloproctology Center/Kurume Hospital, Kurume-City, Japan; Kurume Coloproctology Center/Kurume Hospital, Kurume-City, Japan; Kurume Coloproctology Center/Kurume Hospital, Kurume-City, Japan; Kurume Coloproctology Center/Kurume Hospital, Kurume-City, Japan; Division of Gastroenterology, Department of Medicine, Kurume University School of Medicine, Kurume-City, Japan; Kurume Coloproctology Center/Kurume Hospital, Kurume-City, Japan

**Keywords:** ulcerative colitis, Janus kinase inhibitor, filgotinib, endoscopy, histopathology

## Abstract

**Background/Aims:**

Filgotinib (FIL), a Janus kinase inhibitor, shows clinical efficacy in moderate to severe ulcerative colitis (UC), but no prospective studies have examined endoscopic and histopathological outcomes. This study aimed to evaluate the therapeutic efficacy of FIL in moderate to severe UC using the Partial Mayo Score (PMS), Ulcerative Colitis Endoscopic Index of Severity (UCEIS), and Geboes Histopathology Score (GHS).

**Methods:**

Twenty-two patients with clinically moderate to severe refractory UC were enrolled. Remission was defined as PMS 0, UCEIS 0, and GHS < 2.0 (sigmoid and rectum). Achievement rates were prospectively evaluated at 12, 24, and 52 weeks after FIL initiation compared to baseline.

**Results:**

Among the 22 patients, comprising Biologic-Naïve (BN, *n* = 12) and Biologic-Experienced (BE, *n* = 10) cohorts, achievement rates were highest for PMS 0, followed by UCEIS 0, and lowest for GHS < 2.0. Partial Mayo Score 0 achievement for BN/BE was 75% (*P* = .001)/50% (*P* = .031) at 12 weeks, 75% (*P* = .003)/70% (*P* = .016) at 24 weeks, and 75% (*P* = .002)/70% (*P* = .016) at 52 weeks. Ulcerative Colitis Endoscopic Index of Severity 0 achievement for BN/BE was 58.3% (*P* = .008)/20% (*P* = .016) at 12 weeks, 41.6% (*P* = .019)/40% (*P* = .016) at 24 weeks, and 50% (*P* = .002)/50% (*P* = .016) at 52 weeks. Geboes Histopathology Score < 2.0 (sigmoid) achievement for BN/BE was 25%/0% at 12 weeks, 33.3%/10% at 24 weeks, and 25%/10% at 52 weeks. Geboes Histopathology Score < 2.0 (rectum) achievement for BN/BE was 50%/0% at 12 weeks, 41.6%/20% at 24 weeks, and 33.3%/40% at 52 weeks.

**Conclusions:**

Filgotinib appears to be an effective treatment for UC, demonstrating potential for achieving not only clinical remission but also endoscopic and histopathological remission.

## Graphical Abstract

**Graphical Abstract:**
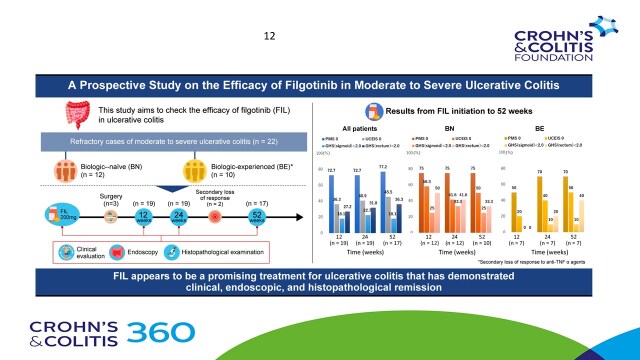

**Key Messages**

**What is known**

- Numerous reports have demonstrated the clinical efficacy of filgotinib, a JAK inhibitor, in moderate to severe ulcerative colitis.
- The balance between eosinophils and neutrophils significantly influences the treatment outcomes in ulcerative colitis. Histopathological remission has emerged as a crucial indicator for improved long-term prognosis.

**What is new here**

- This is the first prospective study to comprehensively evaluate the efficacy of filgotinib from 3 perspectives: clinical, endoscopic, and histopathological outcomes.
- By conducting comparative analyses of endoscopic and histopathological findings from baseline through post-treatment, we elucidated the underlying mechanisms of therapeutic response. Through focused examination of eosinophils and neutrophils using GHS Grade 2A and Grade 2B, we revealed differential suppressive effects of filgotinib on Th1/Th17 and Th2 pathways.
• We identified albumin, hemoglobin, and platelet counts as potential predictive biomarkers for histopathological improvement.

**How can this study help patient care**

- The elucidation of differential effects on Th1/Th17 and Th2 pathways through histopathological evaluation provides valuable guidance for therapeutic strategies in suboptimal responders, such as combination therapy with azathioprine or reintroduction of corticosteroids.
- Pretreatment laboratory parameters (ALB, HGB, PLT) may serve as predictive markers for therapeutic response to filgotinib.
- Our standardized protocol for endoscopic and histopathological evaluation establishes a robust framework for future multicenter studies.

## Introduction

Ulcerative colitis (UC) is a chronic inflammatory bowel disease characterized by continuous inflammation of the mucosa and submucosa from the rectum, accompanied by abdominal symptoms such as diarrhea, abdominal pain, and bloody stools. The prevalence of UC, which primarily affects young individuals, continues to increase.^1^ The disease is characterized by periods of relapse and remission with varying courses, which can complicate treatment strategies.^2^ Consequently, treatment goals for UC, as defined by the STRIDE-II concept,^3^ involve the improvement of clinical symptoms as a short-term objective and endoscopic mucosal healing as a long-term objective. Additionally, histopathological remission has become an important indicator of disease remission.^4^ Histopathological evaluation enables a more precise evaluation of treatment efficacy and helps predict long-term prognosis by assessing microscopic inflammation in the mucosa. Moreover, biopsy specimens obtained from patients with active UC that exhibit high ratios of neutrophil/eosinophil infiltration in the mucosa may indicate potential treatment-refractory cases.^5^ Therefore, evaluating neutrophil/eosinophil infiltration could not only aid in drug assessment but also inform future UC treatment strategies. Comprehensive evaluation,—including clinical symptoms, endoscopic findings, and histopathological assessment,—is crucial as it provides additional information on UC pathophysiology.

Recent years have seen the emergence of various pharmacological treatments for UC, with Janus kinase (JAK) inhibitors gaining particular attention. Janus kinases are enzymes that play a crucial role in intracellular signal transduction from cytokine receptors on the cell membrane.^6^ The pathogenesis of UC involves inflammatory cytokine signaling mediated by the JAK1 complexes.^7^ Among JAK inhibitors, filgotinib (FIL) exhibits strong inhibitory activity against JAK1, achieving therapeutic effects by suppressing the JAK-STAT signaling pathway.^8^ While there are reports on the efficacy of JAK inhibitors, including FIL, these studies primarily focus on clinical evaluations. To date, no prospective studies have examined the endoscopic and histopathological remission induced by FIL. Therefore, this study aimed to comprehensively evaluate the therapeutic efficacy of FIL from 3 perspectives: clinical, endoscopic, and histopathological.

## Materials and Methods

### Description of Participants

**Figure 1.**
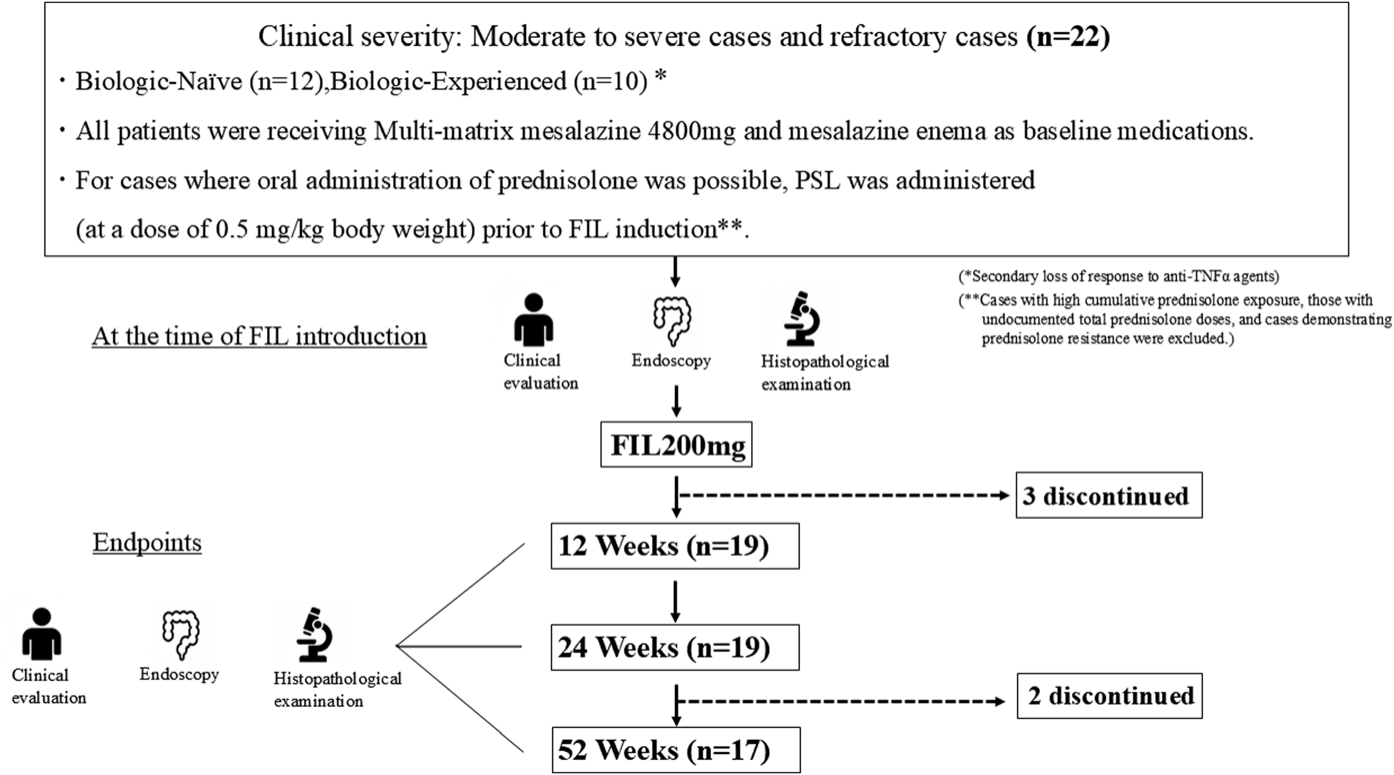
The study included 22 patients with moderate to severe and refractory ulcerative colitis (UC), comprising both Biologic-Naïve (BN) and Biologic-Experienced (BE) groups. Filgotinib (FIL) was initiated in cases of worsening condition or failure to achieve remission with existing treatments. All patients were receiving multi-matrix mesalazine 4800 mg and mesalazine enema as baseline medications. Patients in the BE group had previously received anti-TNFα agents and experienced secondary loss of response. Oral prednisolone (0.5 mg/kg body weight) was administered prior to FIL initiation, excluding cases with high cumulative prednisolone doses, unknown total prednisolone doses, or prednisolone-resistant cases. Clinical, endoscopic, and histopathological evaluations were conducted at baseline (pre-FIL induction) and at weeks 12, 24, and 52 post-induction. Three patients discontinued treatment within the first 12 weeks of FIL administration, and 2 additional patients discontinued between weeks 24 and 52.

This pilot prospective cohort study aimed to elucidate the long-term efficacy and safety of FIL for treating patients with UC. Accordingly, the study included 22 patients with moderate to severe refractory UC who initiated FIL treatment between September 2022 and August 2024 at our institution. The cohort comprised both Biologic-Naïve (BN) and Biologic-Experienced (BE) patients. The clinical severity of UC at the time of FIL initiation was assessed using the severity classification established by the Intractable Inflammatory Bowel Disease Research Group. All patients were receiving multi-matrix mesalazine 4800 mg and mesalazine enema as baseline medications. Filgotinib was administered in cases where existing treatments led to disease exacerbation or failed to achieve clinical remission. All patients in the BE group had previously experienced secondary failure to anti-tumor necrosis factor-α agents (with exposure limited to anti-tumor necrosis factor-α agents only). Oral prednisolone (0.5 mg/kg body weight) was administered prior to FIL initiation, excluding cases with high cumulative prednisolone doses, unknown total prednisolone doses (high cumulative prednisolone doses raised concerns about adverse effects, and unknown total prednisolone doses posed risks of adrenal insufficiency; therefore, we determined that prednisolone could not be administered without careful consideration), or prednisolone-resistant cases. Prednisolone was tapered by 5 mg every 2 weeks, and we adopted a policy of no re-administration after the completion of the tapering regimen. The FIL dose was set at 200 mg once daily, and FIL was administered for 52 weeks. Five patients discontinued treatment during the study period (Figure 1). The sample size for this study was determined based on the following parameters. The expected remission rate was set at 70%, referencing the clinical remission rate (64.6%) of JAK inhibitors in Japanese multicenter studies for UC.^9^ Using a statistical significance level (α) of 0.05 and a power (1-β) of 80%, the required sample size was calculated. Accounting for an anticipated dropout rate of 20% during the 52-week long-term observation period, the final target sample size was established at 22 patients.

### Evaluation Methods

Clinical, endoscopic, and histopathological assessments were conducted at baseline, at 12, 24, and 52 weeks after FIL initiation. The clinical therapeutic effects were evaluated using the Partial Mayo Score (PMS).^10^ The endoscopic therapeutic effect was assessed using the Ulcerative Colitis Endoscopic Index of Severity (UCEIS).^11^ The histopathological therapeutic effect was evaluated using the Geboes Histopathology Score (GHS).^12,13^ (Figure 1). We performed a stratified analysis and visualization of GHS scores by grade, enabling comprehensive evaluation through grade-specific graphical representation.

### Primary Endpoints

The primary endpoints were the rates of remission achievement at 12, 24, and 52 weeks after FIL initiation, defined as follows: Clinical remission: PMS 0, Endoscopic remission: UCEIS 0, Histopathological remission: GHS < 2.0. The treatment continuation rate and safety profile were evaluated at the 52-week time point.

### Secondary Endpoints

Temporal changes in eosinophil (Grade 2A) and neutrophil infiltration (Grade 2B) were assessed using the GHS. At 52 weeks: (1) a comparison of baseline characteristics at FIL initiation between the histological improvement group (GHS < 3.0) and the histological non-improvement group (GHS ≥ 3.0), and (2) a correlation analysis of background factors between these 2 groups, were performed.

### Evaluation Methods for UCEIS and GHS

The UCEIS was assessed through sigmoidoscopy at 12 and 24 weeks after FIL initiation, and total colonoscopy at 52 weeks. Two endoscopists certified by the Japan Gastroenterological Endoscopy Society independently evaluated the UCEIS scores. Although no significant discrepancies in baseline scores were observed, some cases demonstrated subtle scoring inconsistencies after FIL administration. Therefore, we adopted a stringent evaluation approach for such cases (eg, when scores fluctuated between UCEIS 1 and 2, we consistently assigned the higher score of UCEIS 2). For the GHS assessment, biopsy specimens were obtained during all endoscopic examinations at 12, 24, and 52 weeks after FIL initiation. The biopsy sites were standardized and consistently targeted at the sigmoid colon (within 30 cm from the anal verge) and rectum (Rb: within 6 cm from the anal verge), ensuring that the same areas were sampled before and after FIL initiation. Biopsy specimens were obtained from the areas showing the most severe inflammation. This approach was chosen to maintain consistency with endoscopic assessment, as UCEIS scoring is also performed at the sites of maximum inflammatory activity. Histopathological evaluations were conducted independently by 2 pathologists certified by the Japanese Society of Pathology. In cases where there were discrepancies between the assessments of the 2 pathologists, a pathology conference was held to discuss and evaluate the validity of the findings, and a consensus was reached for the final determination. In this study, endoscopic and histopathological evaluations were performed without blinding. However, we attempted to maintain objectivity by having 2 independent specialists perform each assessment.

### Statistical Analysis

Statistical analyses were performed using JMP 16.1.0 software. Data are presented as median values with interquartile ranges (IQRs). Treatment outcomes of FIL in both BN and BE groups were analyzed using the Wilcoxon signed-rank test, with *P* < .05 considered statistically significant. Changes from baseline in both PMS and UCEIS were analyzed using paired *t*-tests with a 95% confidence interval (CI) for both groups (BN: *n* = 12, BE: *n* = 10). Given the small sample sizes (*n* < 30), Student’s *t*-distribution was employed, with 11°C of freedom for the BN group and degrees of freedom based on available cases accounting for dropouts in the BE group. The analysis was conducted at predetermined time points (weeks 12, 24, and 52). This methodological approach ensured robust statistical analysis for both groups despite patient attrition. The aforementioned parametric tests were not applied to GHS analysis, as it is an ordinal rather than a continuous variable. The continuation rate of FIL was evaluated using Kaplan-Meier analysis. At the 52-week time point, comparisons of baseline characteristics at FIL initiation between the GHS < 3.0 and GHS ≥ 3.0 groups were conducted using the Mann–Whitney U test. Correlation analyses between these 2 groups were performed using Spearman’s rank correlation coefficient (r_s_) analysis.

## Results

### Patient Characteristics

Table 1 shows the baseline characteristics of patients at the time of FIL initiation. The cohort comprised 12 males (54.5%) and 10 females (45.5%). Eight patients (36.4%) were prednisolone-dependent, while 5 (22.7%) were prednisolone-resistant. All patients who initiated prednisolone therapy successfully completed the tapering regimen of 5 mg reduction every 2 weeks and achieved complete discontinuation of prednisolone. Prednisolone dependence was defined as the initial clinical response to prednisolone therapy followed by symptom relapse either during steroid tapering or within several months post-tapering. Prednisolone resistance was defined as failure to achieve clinical improvement within 2 weeks despite administration of prednisolone at doses of 0.5 mg/kg. Six patients (27.2%) had received azathioprine therapy, while 16 patients (72.8%) were azathioprine-naïve. The median PMS was 5.5 (IQR 2-8), and the median UCEIS was 6.5 (IQR 4-8), with 11 patients (50.0%) classified as having severe disease. Although 5 patients were classified as having mild disease activity due to the more granular scoring nature of UCEIS, all of these patients had a UCEIS score of 4, representing disease activity at the upper end of the mild category. Furthermore, a UCEIS score of 4 corresponds to a Mayo endoscopic subscore of 2, which is indicative of moderate disease activity. The median C-reactive protein (CRP) level was elevated at 0.505 mg/dL (IQR 0.01-15.43).

**Table 1.** Patient characteristics at baseline

Male	12 (54.5%)	Partial Mayo Score	5.5 (2-8)
**Female**	10 (45.5%)	**Mayo score**	8 (4-11)
**Age**	44.0 (16-74)	Mild (3-5)	2
**Disease duration** (month)	61.0 (5-297)	Moderate (6-10)	18
**Disease extent**		Severe (11-12)	2
Pancolitis	13 (59.1%)	**UCEIS**	6.5 (4-8)
Left-sided colitis	9 (40.9%)	Mild (2-4)	5
Clinical severity		Moderate (5-6)	6
Moderate	21 (95.5%)	Severe (7-8)	11
Severe	1 (4.5%)	**WBC** (/μL)	7320 (3030-15730)
**Prednisolone use**	13(59.1%)	** **Neut (/μL)	4602 (1927-14314)
Dependence	8 (36.4%)	Eosino (/μL)	2.2 (0.1-7.4)
Resistance	5 (22.7%)	**HGB** (g/dL)	13.6 (9.0-16.8)
**Azathioprine use**		**PLT** (× 10^4^/μL)	29.5 (20.6-56.7)
Yes	6 (27.3%)	**ALB** (g/dL)	3.85 (2.3-4.6)
No	16 (72.7%)	**C-reactive protein** (mg/dL)	0.505 (0.01-15.43)

Values are presented as median with interquartile range (IQR).

Abbreviations: ALB, albumin; HGB, hemoglobin; PLT, platelet; UCEIS, Ulcerative Colitis Endoscopic Index of Severity; WBC, white blood cell.

### FIL Initiation at 52 Weeks

**Figure 2.**
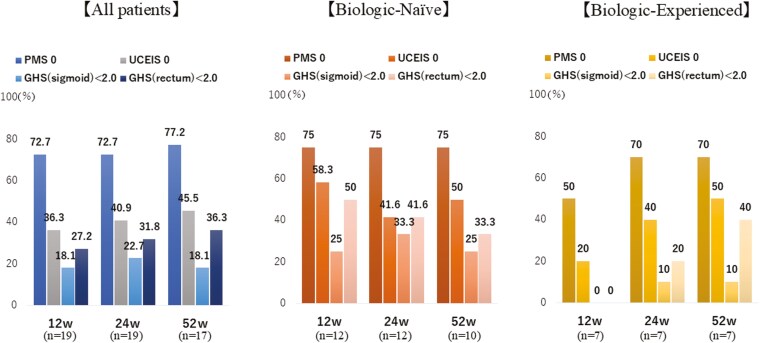
This study presents an overview of all 22 cases and compares the outcomes of the Biologic-Naïve (BN) and Biologic-Experienced (BE) groups from the initiation of filgotinib (FIL) treatment up to 52 weeks. The achievement rates for Partial Mayo Score (PMS) of 0, Ulcerative Colitis Endoscopic Index of Severity (UCEIS) of 0, Geboes Histopathology Score (GHS) < 2.0 in the sigmoid colon, and GHS < 2.0 in the rectum (Rb) are reported at weeks 12, 24, and 52 of treatment.

Among the 22 patients, the achievement rates of PMS 0 were consistently high, reaching 72.7%, 72.7%, and 77.2% at 12, 24, and 52 weeks, respectively. The achievement rates of UCEIS 0 were 36.3%, 40.9%, and 45.5% at 12, 24, and 52 weeks, respectively. The achievement rates of GHS < 2.0 in the sigmoid colon were 18.1%, 22.7%, and 18.1% at 12, 24, and 52 weeks, respectively. In the rectum, these rates were 27.2%, 31.8%, and 36.3% at the same time points. When comparing the BN and BE groups, a higher proportion of patients in the BN group achieved a PMS 0, UCEIS 0, and GHS < 2.0 than the BE group (Figure 2). When comparing our observed clinical remission rates (PMS 0) at week 52 (75% in the BN group and 70% in the BE group) with the expected remission rate of 70%, we found that the outcomes were consistent with or exceeded our hypothesis. Using a one-sample binomial test, the difference between observed and expected rates was not statistically significant (*P* = .89 for the BN group, *P* = .92 for the BE group), though this comparison was limited by our small sample size as this was a pilot study. The primary aim of this pilot study was to comprehensively evaluate clinical, endoscopic, and histopathological outcomes rather than to confirm statistical superiority over historical data. Larger studies will be needed to definitively establish the comparative efficacy of FIL in this setting.

### Treatment Continuation Rate and Safety

**Figure 3.**
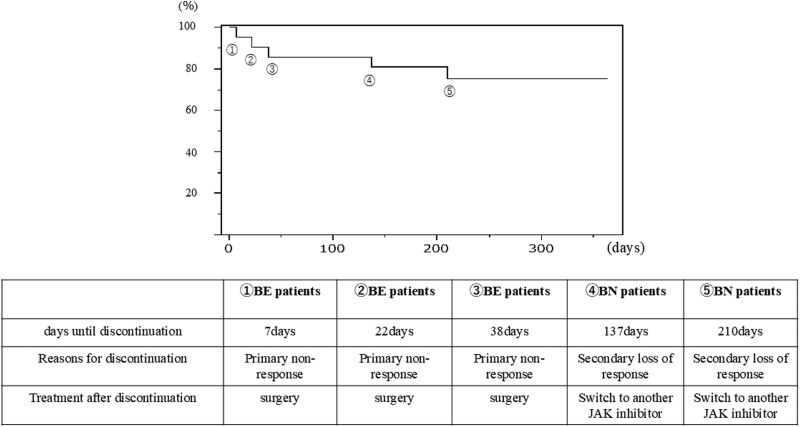
Treatment persistence from FIL initiation through week 52 was illustrated using a Kaplan-Meier analysis. Five patients discontinued therapy during the follow-up period. The detailed information regarding days until discontinuation, reasons for discontinuation, and subsequent treatment after discontinuation has been documented. FIL, filgotinib.

The treatment persistence rate from FIL initiation through week 52 was illustrated using a Kaplan-Meier analysis. The continuation rate at 52 weeks was 77.2% (17 out of 22 patients). Five patients discontinued therapy during the follow-up period, 3 from the BE group and 2 from the BN group. The BE group showed a tendency toward earlier discontinuation. The reasons for discontinuation were Primary non-response in the BE group and Secondary loss of response in the BN group. Regarding treatment after discontinuation, all patients in the BE group underwent surgery, while patients in the BN group switched to another JAK inhibitor (Figure 3). Regarding safety during FIL administration, no deep vein thrombosis or herpes zoster was observed, with dizziness reported in only one patient (1 out of 22 patients, 4.5%).

### Changes in PMS and UCEIS After FIL Initiation

#### Changes in PMS

**Figure 4.**
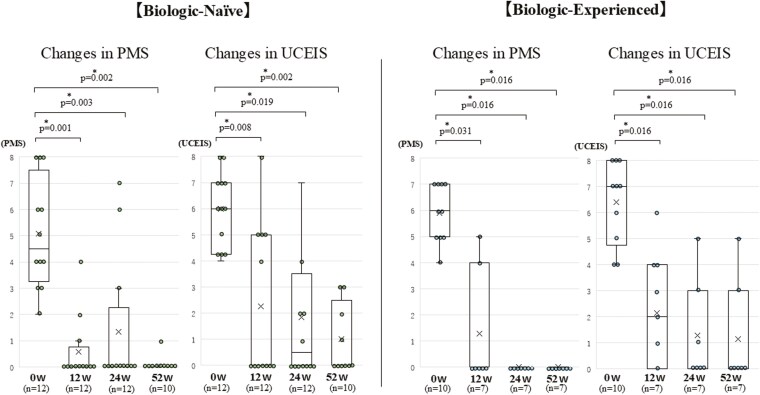
Changes in Partial Mayo Score (PMS) and Ulcerative Colitis Endoscopic Index of Severity (UCEIS) over 52 weeks of filgotinib treatment. This figure illustrates the changes in PMS and UCEIS scores for both Biologic-Naïve (BN) and Biologic-Experienced (BE) patients over the course of 52 weeks of filgotinib treatment. BN group: PMS showed statistically significant improvements. Specifically, significant improvements were observed at 12 weeks (*P* = .001), 24 weeks (*P* = .003), and 52 weeks (*P* = .002). The UCEIS scores showed statistically significant improvements. Specifically, significant improvements were observed at 12 weeks (*P* = .008), 24 weeks (*P* = .019), and 52 weeks (*P* = .002). BE group: PMS showed statistically significant improvements. Specifically, significant improvements were observed at 12 weeks (*P* = .031), 24 weeks (*P* = .016), and 52 weeks (*P* = .016). The UCEIS scores showed statistically significant improvement. Significant improvements were observed at 12 weeks, 24 weeks, and 52 weeks (all *P* = .016). The trend in PMS, Y-axis: PMS score, X-axis: Weeks of treatment (0w, 12w, 24w, 52w). The trend in UCEIS, Y-axis: UCEIS score, X-axis: Weeks of treatment (0w, 12w, 24w, 52w). Statistical significance: *P < .05 (Wilcoxon signed-rank test). Each data point represents the median score. The whiskers indicate the interquartile range. Statistically significant differences from baseline are indicated by asterisks (*). Abbreviation: w, weeks.

Compared to the baseline before FIL initiation, both groups demonstrated statistically significant and clinically meaningful improvements in disease activity as measured by PMS. In the BN group, at 12 weeks after FIL initiation, the mean decrease in PMS from baseline was 4.58 points (95% CI, 3.12-6.04, *P* = .001). At 24 weeks, the mean decrease was 4.17 points (95% CI, 2.56-5.78, *P* = .003), and at 52 weeks, the mean decrease was 4.70 points (95% CI, 3.24-6.16, *P* = .002). In the BE group, at 12 weeks after FIL initiation, the mean decrease in PMS from baseline was 4.57 points (95% CI, 2.89-6.25, *P* = .031). At 24 weeks, the mean decrease was 5.71 points (95% CI, 4.29-7.13, *P* = .016), and this improvement was sustained at 52 weeks with the same mean decrease of 5.71 points (95% CI, 4.29-7.13, *P* = .016) (Figure 4). All patients except for 5 non-responders demonstrated clinical response, with no patients showing deterioration in scores from baseline.

#### Changes in UCEIS

Similarly, when comparing UCEIS to baseline before FIL initiation, both groups exhibited statistically significant and clinically meaningful improvements in disease activity. In the BN group, at 12 weeks after FIL initiation, the mean reduction in UCEIS from baseline was 3.67 points (95% CI, 1.92-5.42, *P* = .008). At 24 weeks, the mean reduction was 3.25 points (95% CI, 1.45-5.05, *P* = .019), and at 52 weeks, the mean reduction reached 4.30 points (95% CI, 2.89-5.71, *P* = .002). In the BE group, at 12 weeks after FIL initiation, the mean reduction in UCEIS from baseline was 4.43 points (95% CI, 2.71-6.15, *P* = .016). At 24 weeks, the mean reduction was 5.86 points (95% CI, 4.14-7.58, *P* = .016), and at 52 weeks, the mean reduction was maintained at 5.00 points (95% CI, 3.28-6.72, *P* = .016) (Figure 4). All patients except for 5 non-responders demonstrated endoscopic response, with no patients showing deterioration in scores from baseline.

### Changes in GHS After FIL Initiation

**Figure 5.**
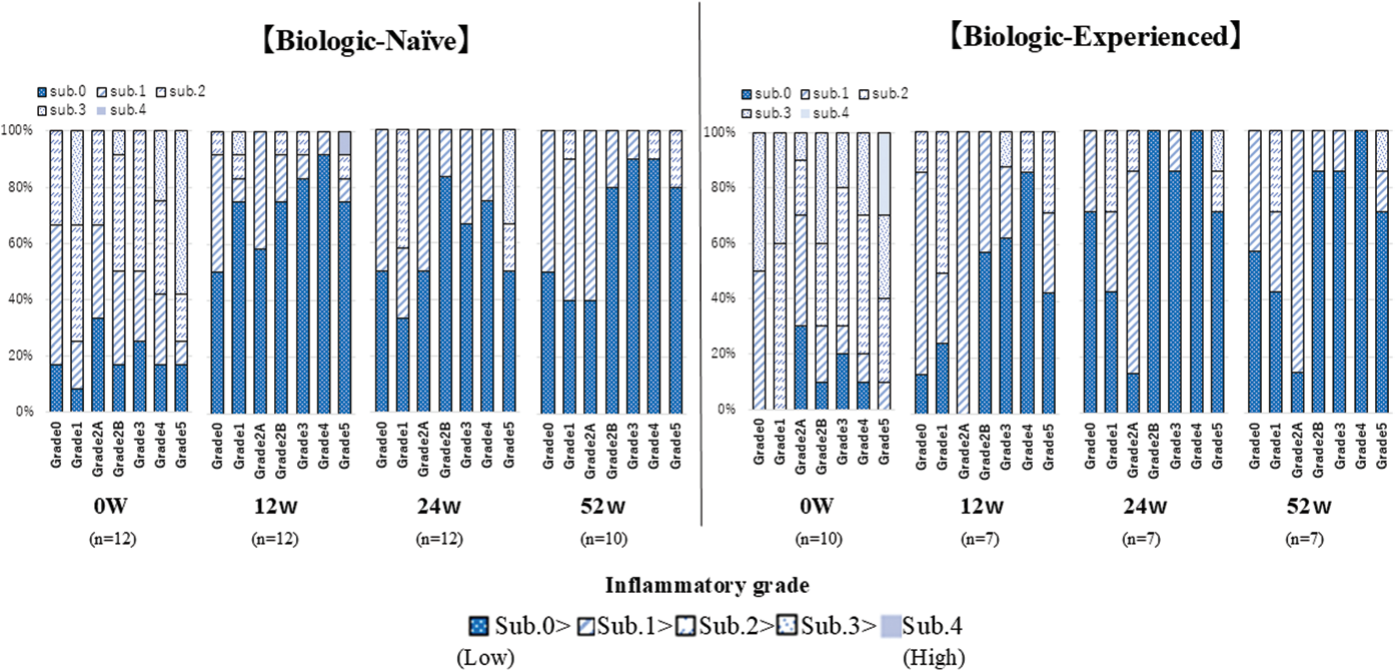
Histopathological changes in sigmoid colon over 52 weeks of filgotinib treatment. Comparison between Biologic-Naïve (BN) and Biologic-Experienced (BE) groups. This figure illustrates the temporal changes in Geboes Histopathology Score (GHS) for the sigmoid colon in BN and BE over 52 weeks of filgotinib treatment. BN group: (*n* = 12 at 0w, 12w, 24w; *n* = 10 at 52w). BE group: (*n* = 10 at 0w; *n* = 7 at 12w, 24w, 52w). Each bar represents the distribution of GHS grades and subgrades at a specific time point. The colors in the stacked bar chart correspond to different grades and subgrades of the GHS. Subgrades are represented within each grade, with lower subgrades indicating less severe inflammation. The progression toward lower grades and subgrades over time indicates histopathological improvement. Y-axis: Percentage of patients. X-axis: Weeks of treatment (0w, 12w, 24w, 52w). Abbreviation; w, weeks.

**Figure 6.**
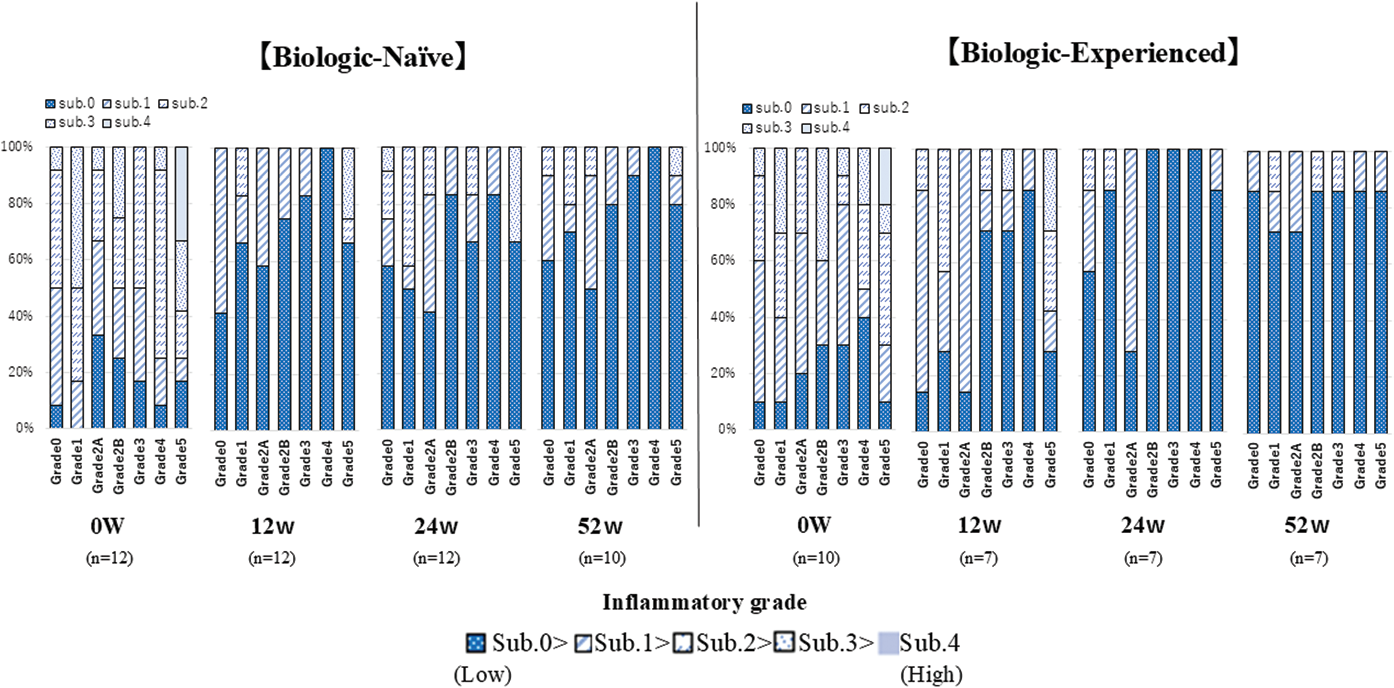
Histopathological changes in rectum over 52 weeks of filgotinib treatment. Comparison between Biologic-Naïve (BN) and Biologic-Experienced (BE) groups. This figure illustrates the temporal changes in Geboes Histopathological Score (GHS) for rectum in BN and BE patients over 52 weeks of filgotinib treatment. BN group: (*n* = 12 at 0w, 12w, 24w; *n* = 10 at 52w). BE group: (*n* = 10 at 0w; *n* = 7 at 12w, 24w, 52w). Each bar represents the distribution of GHS grades and subgrades at a specific time point. The colors in the stacked bar chart correspond to different grades and subgrades of the GHS. Subgrades are represented within each grade, with lower subgrades indicating less severe inflammation. The progression toward lower grades and subgrades over time indicates histopathological improvement. Y-axis: Percentage of patients. X-axis: Weeks of treatment (0w, 12w, 24w, 52w). Abbreviation; w, weeks.

At baseline, the GHS showed a high proportion of subgrade 2 or higher in Grades 1, 2B, 3, 4, and 5. In the sigmoid colon, changes in subgrades within each Grade were observed. At 12 weeks, there was an increase in the proportion of subgrade 0 across all Grades, indicating histopathological improvement of inflammation. By 52 weeks, a further temporal increase in subgrade 0 was observed, which was particularly pronounced in the BN group (Figure 5). Similar changes were observed in the rectum. At 12 weeks, there was an increase in the proportion of subgrade 0 across all Grades, demonstrating histopathological improvement of inflammation. The temporal increase in subgrade 0 continued through 52 weeks, with notable improvements in both groups (Figure 6).

### Changes in Eosinophil (Grade 2A) and Neutrophil (Grade 2B) Levels in GHS

**Figure 7.**
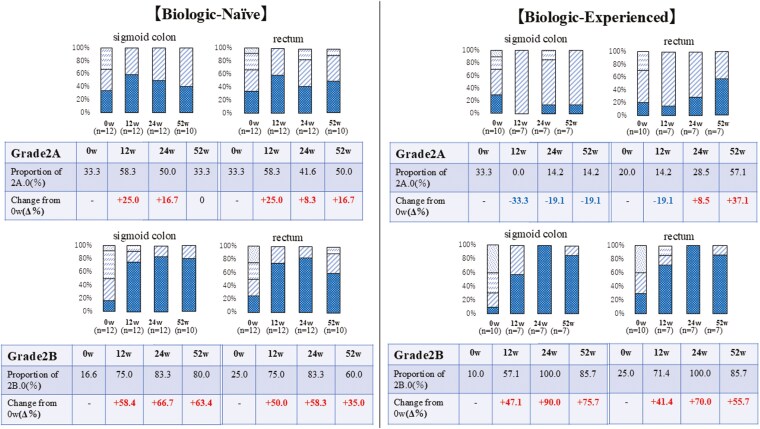
Progression of Geboes Histopathological Score (GHS) Grade 2A and Grade 2B in Biologic-Naïve (BN) and Biologic-Experienced (BE) groups. This figure illustrates the changes in the proportion of patients with GHS Grade 2A and Grade 2B, focusing on subgrades 2A.0 and 2B.0, in both BN and BE groups over 52 weeks of filgotinib treatment. (This figure represents an extraction and juxtaposition of Grade 2A and Grade 2B data from Figure 3.) BN group top row: Sigmoid colon (*n* = 12 at 0w, 12w, 24w; *n* = 10 at 52w), rectum (*n* = 12 at 0w, 12w, 24w; *n* = 10 at 52w) BE group top row: Sigmoid colon (*n* = 10 at 0w; *n* = 7 at 12w, 24w, 52w), rectum (*n* = 10 at 0w; *n* = 7 at 12w, 24w, 52w) Each graph shows: Subgrades are represented within each grade, with lower subgrades indicating less severe inflammation. The progression towards lower grades and subgrades over time indicates histopathological improvement. The study examined the proportions of subgrade 2A.0 and 2B.0 at various time points throughout the observation period. For each subgrade, we analyzed both the percentage of patients achieving these scores at each time point and the change from baseline, expressed as a delta percentage (Δ%). Subgrade .0 within each grade indicates the least severe form of that grade. An increase in the proportion of subgrade 2A.0 or 2B.0 suggests a shift toward less severe histopathological inflammation within that grade. Δ values indicate an improvement (increase in the proportion of subgrade .0) compared to baseline. Y-axis: Percentage of patients. X-axis: Weeks of treatment (0w, 12w, 24w, 52w). Abbreviations: w, weeks Δ, Change from baseline

We extracted and evaluated the proportions of subgrade 2A.0 and subgrade 2B.0 from Grade 2A and Grade 2B, respectively (Figure 7).

#### Grade 2A

In the BN group, the proportions of subgrade 2A.0 in the sigmoid colon were 33.3%, 58.3%, 50.0%, and 33.3% at baseline, 12 weeks, 24 weeks, and 52 weeks after FIL initiation, respectively. In the rectum, these proportions were 33.3%, 58.3%, 41.6%, and 50.0% at the same time points. When measuring changes in the proportion of subgrade 2A.0 using Δ values, a peak increase was observed at 12 weeks. In the BE group, the proportions of subgrade 2A.0 in the sigmoid colon were 33.3%, 0.0%, 14.2%, and 14.2% at baseline, 12 weeks, 24 weeks, and 52 weeks, respectively, whereas in the rectum, these proportions were 20.0%, 14.2%, 28.5%, and 57.1% at the same time points. When measuring changes in the proportion of subgrade 2A.0 using Δ values, an increase was observed only in the rectum.

#### Grade 2B

In the BN group, the proportions of subgrade 2B.0 in the sigmoid colon were 16.6%, 75.0%, 83.3%, and 80.0% at baseline, 12 weeks, 24 weeks, and 52 weeks after FIL initiation, respectively. In the rectum, these proportions were 25.0%, 75.0%, 83.3%, and 60.0% at the same time points. When measuring changes using Δ values, an increase was observed from 12 weeks onward. In the BE group, the proportions of subgrade 2B.0 in the sigmoid colon were 10.0%, 57.1%, 100.0%, and 85.7% at baseline, 12 weeks, 24 weeks, and 52 weeks, respectively. In the rectum, these proportions were 25.0%, 71.4%, 100.0%, and 85.7% at the same time points. When measuring changes using Δ values, an increase was observed from 12 weeks onward. In both the BN and BE groups, the proportion of subgrade 2B.0 increased from 12 weeks and was sustained through 52 weeks in both the sigmoid colon and rectum.

**Figure 8.**
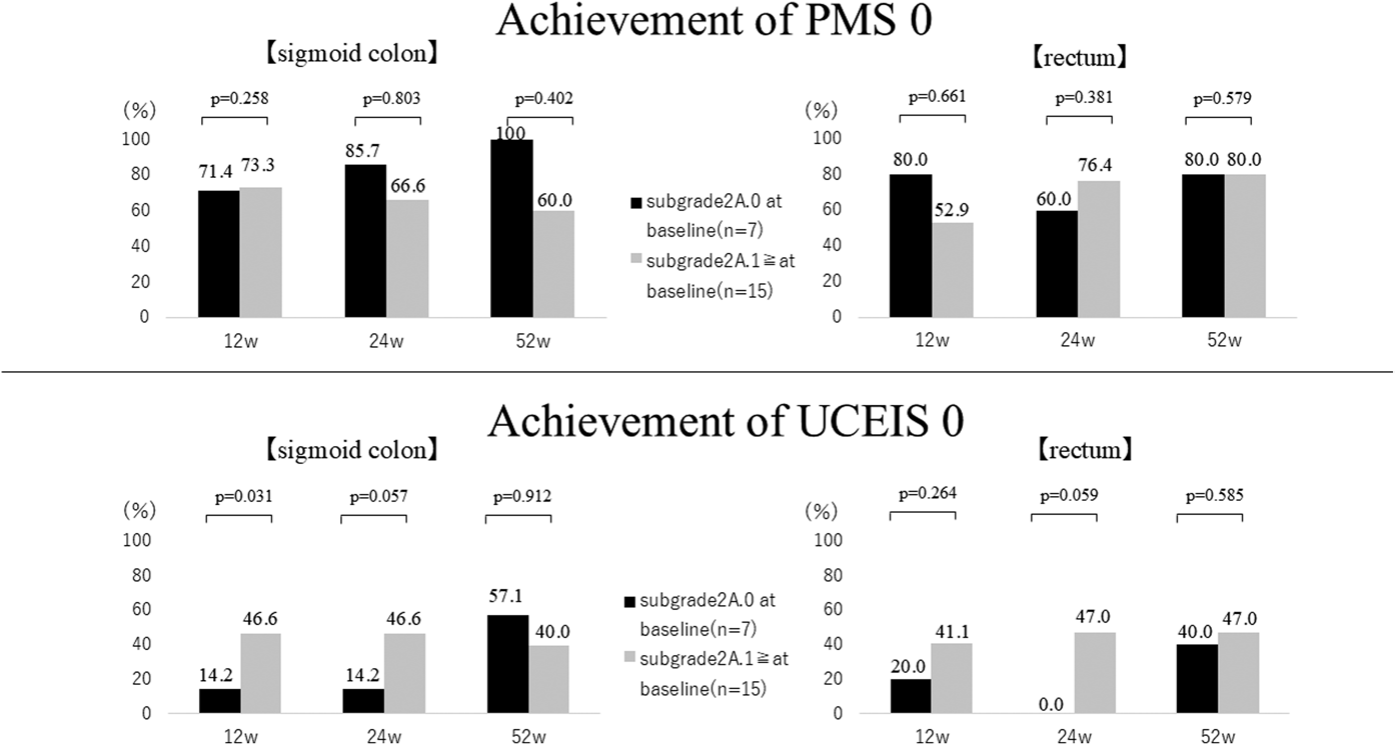
Achievement rates of PMS 0 and UCEIS 0 in sigmoid colon and rectum stratified by baseline subgrade 2A status (subgrade2A.0 vs subgrade2A.1≥). The rates were compared between patients with baseline subgrade2A.0 (*n* = 7) and those with baseline subgrade2A.1 ≥ (*n* = 15) at 12, 24, and 52 weeks. Statistical significance was determined using Wilcoxon test with *P* < .05 considered statistically significant. PMS, Partial Mayo Score; UCEIS, Ulcerative Colitis Endoscopic Index of Severity. Y-axis: Achievement rates (%). X-axis: Time points of evaluation (12w, 24w, 52w). Abbreviation: w, weeks.

**Figure 9.**
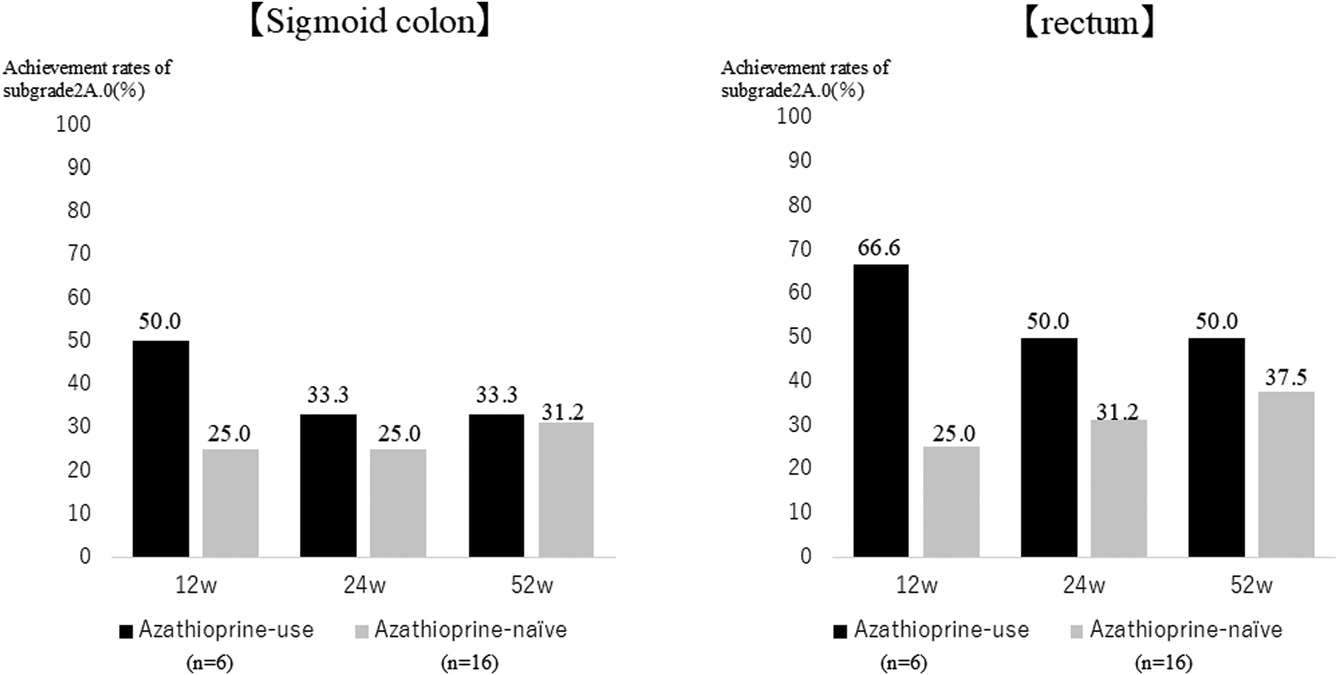
Achievement rates of sub2A.0 at 12, 24, and 52 weeks after filgotinib initiation in azathioprine use and azathioprine-naïve patients. Y-axis: Achievement rates of sub2A.0 (%). X-axis: Weeks after filgotinib initiation (12w, 24w, 52w). Abbreviation: w, weeks.

As demonstrated in Figure 7, the temporal changes in Δ values for Grade2A.0 did not show consistent improvement, suggesting potentially limited therapeutic efficacy in patients with Subgrade 2A.0. Therefore, regardless of BN or BE status, we stratified patients based on their baseline histological severity (Subgrade 2A.0 vs Subgrade 2A.1≥) to evaluate the therapeutic efficacy of FIL. Statistical analysis was performed using the Wilcoxon signed-rank test, with statistical significance set at *P* < .05 (Figure 8). Results demonstrated no significant differences in therapeutic response between these subgroups. Although no significant differences in therapeutic response were observed between these subgroups, the achievement rate of PMS0 was higher in subgrade 2A.0, while the achievement rate of UCEIS0 was higher in subgrade 2A.1. Furthermore, we investigated the potential association between azathioprine administration and achievement of Subgrade 2A.0. Comparative analysis between azathioprine-treated patients (*n* = 6) and azathioprine-naïve patients (*n* = 16) in both sigmoid colon and rectum demonstrated that patients receiving azathioprine therapy achieved significantly higher rates of Subgrade 2A.0 compared to azathioprine-naïve subjects (Figure 9).

### Comparison of (1) Baseline Characteristics and (2) Correlations Between Histological Improvement (GHS < 3.0) and Non-Improvement (GHS ≥ 3.0) Groups at 52 Weeks

We divided the patients into 2 groups based on their GHS at 52 weeks: GHS < 3.0 (histopathological improvement) and GHS ≥ 3.0 (histopathological non-improvement), for both the sigmoid colon and rectum. Subsequently, we compared baseline characteristics at FIL initiation between these groups. In the sigmoid colon, hemoglobin (HGB) levels showed a significant difference between the GHS < 3.0 and GHS ≥ 3.0 groups (*P *= .008). In the rectum, significant differences were observed in HGB levels (*P* = .048) and platelet (PLT) counts (*P* = .032). Further correlation analysis of these parameters revealed that in the sigmoid colon, albumin (ALB) and HGB levels showed significant positive correlations (ALB: r_s_ = 0.46, HGB: r_s_ = 0.58), while the PLT count showed a significant negative correlation (PLT: r_s_ = −0.44) with histopathological improvement (Table 2). Similarly, in the rectum, ALB and HGB levels demonstrated significant positive correlations (ALB: r_s_ = 0.43, HGB: r_s_ = 0.50), while the PLT count showed a significant negative correlation (PLT: r_s_ = −0.44) with histopathological improvement (Table 3).

**Table 2.** Baseline characteristics: Comparison between histopathological responders and non-responders in sigmoid colon at week 52 and their correlation with treatment outcome.

	Response(GHS < 3.0) *n* = 11	Non-response(GHS ≥ 3.0) *n* = 11	*P*-value^a^	r_s_^b^
Age	50 (32-64)	39 (16-73)	.128	0.32
Sex				
Male	6	5		
Female	4	7		
BMI	22.6 (18.6-30.9)	21.08 (16.7-33.8)	.210	0.14
Disease extent				
Left-sided colitis	6	3		
Pancolitis	4	9		
Disease duration(month)	93 (21-297)	39.5 (7-189)	.146	0.15
Prednisolone use				
Dependence	7	8		
Resistance	2	3		
None	1	1		
Duration of steroid treatment prior to FIL initiation(month)	38 (31-66)	44.5 (28-240)	.519	0.35
Use of mesalazine enema use	9	6		
Use of immunosuppressants	1	3		
Biologic-naïve	6	6		
Biologic-experienced	4	6		
ALB	4.0 (3.8-4.4)	3.6 (2.3-4.6)	.059	0.46
WBC	7425 (4360-12380)	6855 (3030-15730)	.895	−0.08
HGB	14.2 (11.3-16.8)	10.9 (9.0-14.9)	.008	0.58
PLT	26.1 (22.3-41.6)	34.9 (20.6-56.7)	.121	−0.44
CRP	0.35 (0.01-14.32)	0.81 (0.01-15.43)	.355	−0.30
PMS score	5 (3-8)	6 (2-8)	.640	−0.23
UCEIS score	6 (4-8)	7 (4-8)	.379	−0.17

Values are presented as median with interquartile range (IQR).

^a^Mann-Whitney U test.

^b^Spearman’s rank correlation coefficient.

Abbreviations: ALB, albumin; BMI, body mass index; CRP, C-reactive protein; HGB, hemoglobin; PLT, platelet; PMS, Partial Mayo Score; UCEIS, Ulcerative Colitis Endoscopic Index of Severity; WBC, white blood cell.

**Table 3.** Baseline characteristics: Comparison between histopathological responders and non-responders in rectum at week 52 and their correlation with treatment outcome.

	Response(GHS < 3.0) *n* = 12	Non-response(GHS ≥ 3.0) *n* = 10	*P*-value^a^	r_s_^b^
Age	46.5 (32-74)	39 (16-73)	.552	0.14
Sex				
Male	7	5		
Female	5	5		
BMI	22.1 (17.5-31.9	21.5 (16.7-33.8)	.644	0.02
Disease extent				
Left-sided colitis	7	6		
Pancolitis	5	4		
Disease duration(month)	76.5 (21-189)	54 (7-297)	.552	0.15
Prednisolone use				
Dependence	9	6		
Resistance	1	3		
None	2	1		
Duration of steroid treatment prior to FIL initiation (month)	42.5 (28-69)	35.5 (28-240)	.732	0.08
Use of mesalazine enema use	9	6		
Use of immunosuppressants	2	2		
Biologic-naïve	7	5		
Biologic-experienced	5	5		
ALB	4 (2.9-4.4)	3.75 (2.8-4.5)	.105	0.43
WBC	7297 (3030-12380)	6855 (4360-12150)	.724	−0.08
HGB	14.2 (9-16.8)	11.2 (9.4-14.3)	.048	0.50
PLT	25.3 (20.6-46.1)	34.95 (26.8-56.7)	.032	−0.44
CRP	0.42 (0.01-15.43)	0.815 (0.03-3.1)	.409	−0.06
PMS	5.5 (4-8)	6 (3-8)	1	−0.16
UCEIS	6 (4-8)	7 (4-8)	1	−0.15

Values are presented as median with interquartile range (IQR).

^a^Mann-Whitney U test.

^b^Spearman’s rank correlation coefficient.

Abbreviations: ALB, albumin; BMI, body mass index; CRP, C-reactive protein; HGB, hemoglobin ; PLT, platelet; PMS, Partial Mayo Score; UCEIS, Ulcerative Colitis Endoscopic Index of Severity; WBC, white blood cell.

## Discussion

Ulcerative colitis is characterized by cycles of relapse and remission. The underlying pathology involves a complex interplay of cytokines primarily mediated by Th1, Th17, and Th2 cells, contributing to the multidimensional nature of UC.^14^ This complexity often leads to varied treatment courses, potentially complicating therapeutic strategies.^15^ Even when clinical and endoscopic remission is achieved, symptom recurrence can occur. Therefore, histopathological evaluation serves as a valuable tool in assessing disease activity and treatment efficacy over time in UC.^15^ Several histopathological scoring systems exist for UC, including the GHS, Robarts Histopathology Index, and Nancy Index. Among these, the GHS is most widely used due to its specificity and comprehensibility.^16–18^ The GHS allows for individual assessment by grade, with additional subgrades enabling fine-tuned evaluation.^12,13^ From a histopathological perspective, eosinophil concentrations reflect Th2 activity, while neutrophil concentrations reflect Th1 and Th17 activity. Consequently, the GHS can be considered to reflect Th2 (Grade 2A), Th1, and Th17 (Grades 2B, 3, 4, and 5) responses.^19,20^ The relationship between GHS and cytokines has been comprehensively described by Sugimoto.^20^ While many studies define histopathological remission as GHS < 3.0, the European Crohn’s and Colitis Organisation guidelines specify cutoff values for histopathological assessment using GHS, with histopathological remission defined as GHS < 2.0 and histopathological improvement as GHS < 3.0.^18^ Therefore, in this study, we defined histopathological remission as GHS < 2.0 and histopathological improvement as GHS < 3.0.

As treatment strategies become more complex, JAK inhibitors have emerged as a new therapeutic option. Among these, FIL has shown promising results in real-world data for clinical remission. In the international phase IIb/III SELECTION trial, following a 10-week induction period and a 48-week maintenance period (total 58 weeks), the PMS achievement rates were reported as 37.2% in the BN group and 23.8% in the BE group.^21^ Gros et al. reported achievement rates of PMS < 2 as 72% and 76% at 12 and 24 weeks, respectively.^22^ Furthermore, Akiyama et al. reported achievement rates of PMS < 1 as 47%, 55.8%, and 64.6% at 10, 26, and 58 weeks, respectively.^9^ Although the SELECTION trial incorporated endoscopic and histopathological evaluations, we implemented a more stringent assessment protocol by employing the UCEIS and establishing a clearly defined threshold of GHS < 2.0. Our study design distinctly differentiated itself from the SELECTION trial through its comprehensive histopathological evaluation approach, which involved baseline tissue sampling followed by comparative analyses at weeks 12, 24, and 52. This systematic methodology enabled us to acquire more profound insights into the histopathological background of both disease progression and treatment response. However, except for the SELECTION trial, no reports have evaluated FIL efficacy using both the UCEIS and GHS, as in our study. This is the first report to assess FIL from 3 perspectives: clinical, endoscopic, and histopathological. Our study demonstrates the importance of evaluating not only clinical remission but also endoscopic and histopathological remission. In this study, the achievement rates of remission following FIL administration were observed in descending order: PMS 0, UCEIS 0, and GHS < 2.0. The findings demonstrate that clinical improvement does not necessarily translate directly to endoscopic or histopathological remission, underscoring the critical importance of comprehensive evaluation through both endoscopic and histopathological assessments. In our protocol, histopathological evaluation using GHS was conducted at 2 distinct anatomical locations: the sigmoid colon and rectum. Notably, given that all patients received concurrent topical therapy, the achievement rate of GHS < 2.0 was superior in rectal specimens, highlighting the potential significance of combination therapy with topical agents in optimizing treatment outcomes. This comprehensive approach aligns with the short-term and long-term goals outlined in STRIDE-II,^3^ and we believe it is crucial for accurately assessing the therapeutic efficacy of FIL.

The GHS is unique among histopathological evaluation methods in its reflection of both eosinophil and neutrophil infiltration. Our study design focused on this aspect, allowing for a prospective evaluation of both eosinophilic and neutrophilic infiltration. Neutrophils are typically scarce in the normal intestinal epithelium. However, in UC, the presence of neutrophils within the intestinal epithelium is an indicator of active inflammation. A reduction or disappearance of neutrophils in the intestinal epithelium is expected to lead to histopathological improvement.^23^ Conversely, while eosinophils are naturally abundant in the intestinal epithelium, their role in the mucosa of refractory UC cases remains unclear.^24,25^ Therefore, we considered the evaluation of Grade 2A and Grade 2B in the GHS crucial for the histopathological assessment of FIL efficacy. In our study, both BN and BE groups showed improvement in subgrade 2B.0 of Grade 2B from 12 weeks after FIL administration, indicating a decrease in neutrophil infiltration. This suggests that FIL may have an early suppressive effect on Th1 and Th17 responses in the mucosal epithelium. However, the improvement in subgrade 2A.0 of Grade 2A was relatively less pronounced. Considering the eosinophil infiltration suppression effect of FIL observed in this study, it can be inferred that FIL’s Th2 suppression effect may be less potent than its Th1 and Th17 suppression effects. Consequently, in cases where Th2 becomes the primary cytokine pathway (FIL-ineffective cases), a combination therapy of FIL with azathioprine or reintroduction of prednisolone might be effective for Th2 suppression.^19,26^ In cases where Th2 emerges as the predominant cytokine pathway (FIL-ineffective cases), previous literature has documented the potential efficacy of combination therapy with azathioprine or prednisolone reintroduction for Th2 suppression. The results from our current study, as demonstrated in Figures 8 and 9, provide additional evidence supporting the potential effectiveness of azathioprine combination therapy in Th2 pathway suppression. While these findings may support the hypothesis that FIL has limited regulatory effects on Th2 cytokines, our study’s relatively small sample size necessitates further investigation to definitively establish the relationship between the degree of Th2 cytokine involvement and therapeutic outcomes. Given the observed disparities in achievement rates between PMS0 and UCEIS0 when stratifying patients by subgrade 2A.0 and subgrade 2A.1, the therapeutic efficacy of FIL specifically within Grade 2A populations warrants further investigation and remains a critical area for future research. This differential response pattern in endoscopic outcome measures within Grade 2A subclassifications highlights the complexity of evaluating therapeutic responses and necessitates more detailed exploration to optimize treatment strategies. This prospective evaluation, including UCEIS and GHS, has enabled us to demonstrate the novelty and utility of FIL in UC treatment.

Given the low achievement rate of GHS < 2.0 in our study, developing strategies to attain this goal has emerged as a critical challenge. Upon examining the process leading to GHS < 2.0 achievement, we identified GHS < 3.0 as an important intermediate target. Consequently, we investigated the background factors associated with achieving GHS < 3.0 at 52 weeks. Regarding factors reflecting clinically active UC and blood test results, Uchihara et al. reported the utility of CRP, PLT, and ALB levels,^27^ while Seo et al. emphasized the usefulness of HGB and ALB levels.^28^ Furthermore, Akiyama et al. identified the PLT count as a valuable predictor of treatment response.^9^ While there are no previous reports on predictive factors for histopathological improvement in UC treated with FIL, our study identified ALB, HGB, and PLT levels as potential predictors of histopathological improvement. These findings are corroborated by previous studies. Higher levels of ALB and HGB reflect improvements in the general condition and anemia, respectively, while lower PLT values suggest a reduction in the inflammatory response. Therefore, we propose that using ALB, HGB, and PLT values as indicators for treatment before FIL administration may increase the likelihood of achieving histopathological remission. This approach could potentially guide treatment strategies aimed at improving histopathological outcomes in UC patients treated with FIL.

In the treatment strategy for UC, there is currently no specific test to accurately determine individual patient cytokine profiles. When administering FIL for UC, we propose conducting histopathological evaluations using the GHS. By observing changes in GHS Grade 2A and Grade 2B, we can infer alterations in Th1, Th2, and Th17 cytokine profiles, assess treatment efficacy, and devise treatment strategies using ALB, HGB, and PLT levels as indicators. This approach may lead not only to clinical remission but also to endoscopic and histopathological remission, representing a significant advancement in UC management. However, we acknowledge the limitation of being a single-center study with a small patient cohort, which may restrict the generalizability of our results. Therefore, we strategically utilized our single-center setting by conducting this research as a pilot study. Our emphasis on histopathological evaluation was motivated by 2 key factors: the current limited implementation of histopathological assessments in Japan, and the absence of comparative histopathological analyses between baseline and post-therapeutic intervention states. A notable strength of our study is the histopathological evaluation conducted by 2 pathologists and the standardization of biopsy procedures, ensuring consistency in evaluation criteria. This maximized the advantages of a single-center study. While our current study focused exclusively on the histopathological evaluation of FIL without comparison to other therapeutic agents, we believe that this FIL-derived data could serve as a foundation for comparative GHS evaluations with other treatments under the same research design. This approach would potentially enable more profound insights into UC treatment strategies. In the current therapeutic landscape for UC, given the absence of established biomarkers for determining specific therapeutic agents or predicting treatment outcomes, histopathological evaluation may emerge as a pivotal strategy in therapeutic decision-making. Looking ahead, we believe this study could contribute to the standardization of endoscopic and histopathological evaluations in future multicenter studies. To achieve this, the following would be necessary: standardization of endoscopic observation sites, unification of biopsy collection site procedures, and participation of pathologists. If these conditions are met, multicenter evaluations would become feasible, potentially contributing to the establishment of new treatment strategies for UC.

## Conclusion

Filgotinib has demonstrated efficacy not only in achieving clinical and endoscopic remission but also in attaining histopathological remission, including improvement in neutrophil infiltration. Therefore, FIL can be considered an effective treatment for moderate to severe UC. Moving forward, further analysis of long-term outcomes is warranted. Additionally, a comprehensive approach combining endoscopic and histopathological evaluations is expected to be valuable in assessing disease activity and predicting prognosis in UC patients.

## Data Availability

The data that support the findings of this study are not publicly available due to their containing information that could compromise the privacy of research participants but are available from the corresponding author (Y.S.) upon reasonable request.
